# Prevalence of the Catatonic Syndrome in an Acute Inpatient Sample

**DOI:** 10.3389/fpsyt.2014.00174

**Published:** 2014-12-03

**Authors:** Mirella Stuivenga, Manuel Morrens

**Affiliations:** ^1^Collaborative Antwerp Psychiatric Research Institute (CAPRI), University of Antwerp, Antwerp, Belgium; ^2^Psychiatric Center Brothers Alexians, Boechout, Belgium

**Keywords:** catatonia, psychomotor, acute psychiatric admissions, classification, schizophrenia, mood disorders

## Abstract

**Objective:** In this exploratory open label study, we investigated the prevalence of catatonia in an acute psychiatric inpatient population. In addition, differences in symptom presentation of catatonia depending on the underlying psychiatric illness were investigated.

**Methods:** One hundred thirty patients were assessed with the Bush–Francis Catatonia Rating Scale (BFCRS), the Positive and Negative Syndrome Scale, the Young Mania Rating Scale, and the Simpson–Angus Scale. A factor analysis was conducted in order to generate six catatonic symptom clusters. Composite scores based on this principal component analysis were calculated.

**Results:** When focusing on the first 14 items of the BFCRS, 101 patients (77.7%) had at least 1 symptom scoring 1 or higher, whereas, 66 patients (50.8%) had at least 2 symptoms. Interestingly, when focusing on the DSM-5 criteria of catatonia, 22 patients (16.9%) could be considered for this diagnosis. Furthermore, different symptom profiles were found, depending on the underlying psychopathology. Psychotic symptomatology correlated strongly with excitement symptomatology (*r* = 0.528, *p* < 0.001) and to a lesser degree with the stereotypy/mannerisms symptom cluster (*r* = 0.289; *p* = 0.001) and the echo/perseveration symptom cluster (*r* = 0.185; *p* = 0.035). Similarly, manic symptomatology correlated strongly with the excitement symptom cluster (*r* = 0.596; *p* < 0.001) and to a lesser extent with the stereotypy/mannerisms symptom cluster (*r* = 0.277; *p* = 0.001).

**Conclusion:** There was a high prevalence of catatonic symptomatology. Depending on the criteria being used, we noticed an important difference in exact prevalence, which makes it clear that we need clear-cut criteria. Another important finding is the fact that the catatonic presentation may vary depending on the underlying pathology, although an unambiguous delineation between these catatonic presentations cannot be made. Future research is needed to determine diagnostical criteria of catatonia, which are clinically relevant.

## Introduction

Catatonia is a psychomotor symptom cluster characterized by a heterogeneous group of mental, motor, vegetative, and behavioral signs. The recognition of catatonia is essential since it is a syndrome that can be effectively and rapidly relieved in most cases. Whereas, the pathophysiology of catatonia is still unknown, it is clear that the psychomotor syndrome results from many etiologies (1).

Although some critics have suggested the syndrome is much more uncommon than a century ago or may even be disappearing, catatonia is still highly prevalent (2). Whereas early investigators reported catatonia in 20–50% of the schizophrenic patients (3, 4), contemporary literature demonstrates the presence of catatonia in 4–15% of schizophrenia patients (5–8). In acutely ill psychiatric inpatients higher estimates are reported, ranging between 5 and 20% (9, 10).

Most recently, the DSM-5 rightfully loosened the association between schizophrenia and catatonia that was predominant in its preceding editions and now recognizes that catatonia can be induced by different disorders (11). In the study of Pommepuy and Januel, including 607 catatonic patients, there was an average of 30.9% of all patients with a primary diagnosis of schizophrenia, whereas 43% of the patients had a mood disorder (12). The review of Caroff and colleagues shows similar results (13). Among patients with a mood disorder, catatonia can be seen in patients with a bipolar disorder with a percentage of 17–47% in mania and 0–20% in patients with a depressive episode (14, 15). In a study including patients with an unipolar depressive disorder 20% of the patients met the criteria for catatonia (16).

There are reasons to believe that the profile of catatonic symptomatology may depend on the underlying pathology (15, 17). Krüger and colleagues demonstrated that catatonia in schizophrenia was mainly characterized by abnormal movements, stereotypies, mannerisms, catalepsy, negativism, automatic obedience, and waxy flexibility, whereas, catatonic excitation was more associated with mania and catatonic inhibition more with depression (15). This notion is very intriguing since it can both have diagnostical and therapeutical implications and give clues toward future research on the underlying pathophysiology of the psychomotor syndrome.

In the present study, prevalence of catatonia in an acute psychiatric inpatient population was investigated. In addition, differences in symptom presentation of catatonia depending on the underlying psychiatric illness were investigated.

## Materials and Methods

### Study design

In an exploratory open label study design, each patient admitted to a psychiatric intensive ward during a period of 12 months was assessed for catatonic and clinical symptomatology. The patients admitted to this department were experiencing the most acute phase of a mental illness. The department is for men and women over the age of 18 year who require a period of psychiatric intensive care. The assessments were conducted on the first day of admission in the hospital. There were no exclusion criteria for participation. All of the 130 patients who were admitted to the psychiatric intensive ward were included in the study.

### Participants

A total group of 130 patients (female: *n* = 50; 38.5%) were tested after admission on an acute psychiatric enclosed ward. The mean age was 40.5 years (SD = 13.9; range 18–76). More than half of our patient group had a psychotic illness as a primary illness (*n* = 67; 51.5%) including 26 patients (20.0%) with schizophrenia (amongst which 3 patients with a diagnosed catatonic subtype) and 35 patients with a psychotic illness not otherwise specified (26.9%). The second most common primary diagnosis (*n* = 16; 12.3%) was a bipolar disorder, followed by substance abuse disorders (*n* = 14; 10.8%). Major depressive disorder was the main diagnosis in six patients (4.6%). Similarly, six patients received a diagnosis of personality disorder (4.6%).

Antipsychotics were taken by 56.9% of the patients (*n* = 74). Twenty-six patients (20.0%) took at least 1 first generation antipsychotic (FGA), whereas 64 patients (49.2%) took a second generation antipsychotic (SGA), 4 patients were taking lithium (3.1%), whereas 12 patients took anti-epileptics (9.2%) at the time of testing. Antidepressants were administered to 30.8% of the patients at the time of testing [SSRI (*n* = 17); SNRI (*n* = 10); TCA (*n* = 2); and others (*n* = 5)]. Finally, 40% of the patients were taking benzodiazepines (n = 52) and 6 patients took an anticholinergic agent (4.6%).

### Clinical assessment

All patients were assessed with the Bush–Francis Catatonia Rating Scale (BFCRS) (18), the Positive and Negative Syndrome Scale (PANSS) (19), the Young Mania Rating Scale (YMRS), and the Simpson–Angus Scale (SAS).

The BFCRS is used to recognize and score catatonic signs and symptoms. It measures the severity of 23 catatonic signs. By scoring the first 14 items of the BFCRS, the instrument can be used as a screening tool. If two or more of the BFCRS signs are present, the presence of catatonia can be considered. Items of the BFCRS are scored on a 0–3 point scale. The PANSS is a widely used medical scale for measuring symptom severity of patients with schizophrenia. Scores ranging from 1 to 7 are given on 30 different symptoms in three subscales (positive scale 7 items, negative scale 7 items, general psychopathology scale 16 items), with total score ranging from 30 to 210. In order to measure depressive symptoms we used a depression-subscale of the PANSS (PANSS-dep) including items depression, anxiety and guilt feelings. The YMRS is a rating scale to assess manic symptoms. The scale has 11 items and is based on the patient’s subjective report of his or her clinical condition over the previous 48 h. Additional information is based upon clinical observations made during the course of the clinical interview. The SAS is used to measure extrapyramidal symptoms. It is composed of 10 items and signs.

## Results

### Catatonia symptomatology

Catatonic symptomatology was highly prevalent in our patient sample. When focusing on the first 14 items of the BFCRS, which are suggested for using the instrument as a screening tool, 101 patients (77.7%) had at least 1 symptom scoring 1 or higher, whereas 66 patients (50.8%) had at least 2 symptoms. Interestingly, when focusing on the DSM-5 criteria of catatonia (at least 3 out of 12 selected symptoms), 22 patients (16.9%) fulfill the diagnostic criteria, which still implied a high prevalence rate, but drastically lower than when using the BFCRS-criteria, and interestingly and unexpectedly, also lower than with the DSM-IV-TR criteria (see Table 1).

**Table 1:** **Prevalence of catatonia in an acute psychiatric patient sample according to different criteria**.

	DSM-IV (20)	DSM-V (11)	BFCRS (18)	Fink and Taylor (21, 22)
Psychotic disorder	19 (28.4%)	14 (20.9%)	48 (71.6%)	9 (13.4%)
Mood disorder	7 (31.8%)	5 (22.7%)	17 (77.3%)	5 (22.7%)
Substance use disorder	1 (7.1%)	0 (0%)	3 (21.4%)	0 (0%)
Another diagnosis	5 (18.5%)	3 (11.1%)	14 (51.9%)	2 (7.4%)
Total patient group	32 (24.6%)	22 (16.9%)	82 (63.1%)	16 (12.3%)

In our patient sample, the most prevalent catatonic symptoms were excitement (*n* = 49; 37.7%), perseveration (*n* = 32; 24.6%), impulsivity (*n* = 31; 23.8%), and verbigeration (*n* = 31; 23.8%), whereas, a grasp reflex or waxy flexibility could not be observed in any of the patients. Similarly, catatonic symptoms such as mitgehen (*n* = 3; 2.3%), gegenhalten (*n* = 2; 1.5%), or ambitendency (*n* = 3; 2.3%) could only seldomly be observed (see Table 2).

**Table 2:** **Scores on the individual items of the BFCRS**.

	Score = 0 (absent symptom)	Score = 1	Score = 2	Score = 3	Patients with symptom (*N*)
Excitement	81	35	14	0	49
Immobility/stupor	107	18	5	0	23
Mutism	117	4	6	3	13
Staring	101	22	5	2	29
Posturing/catalepsy	112	11	4	3	18
Grimacing	119	11	0	0	11
Echopraxia/echolalia	126	3	1	0	4
Stereotypy	104	19	6	1	26
Mannerisms	114	7	7	2	16
Verbigeration	99	17	12	2	31
Rigidity	115	13	2	0	15
Negativism	124	5	1	0	6
Waxy flexibility	130	0	0	0	0
Withdrawal	107	12	6	5	23
Impulsivity	99	13	18	0	31
Automatic obedience	121	4	5	0	9
Mitgehen	127	0	0	3	3
Gegenhalten	128	0	0	2	2
Ambitendency	127	0	0	3	3
Grasp reflex	130	0	0	0	0
Perseveration	98	0	0	32	32
Combativeness	112	15	2	1	18
Autonomic abnormality	116	13	1	0	14

A factor analysis (Principal Component Analysis, varimax rotation) was conducted in order to generate catatonic symptom clusters. Given that items grasp reflex and waxy flexibility had a zero variance, these items were excluded from the analysis. This yielded six symptom clusters (see Table 3): a negative factor including immobility/stupor, mutism, staring, posturing, rigidity, negativism, withdrawal, gegenhalten, and ambitendency; a stereotypy/mannerism factor including stereotypy, mannerisms, and mitgehen; an echo/perseveration factor including echophenomena, verbigeration, and perseveration; an excitement factor encompassing items excitement, impulsivity, and combativeness; a grimacing factor only including that specific item, and finally, an autonomic factor including autonomic abnormalities and, strangely, automatic obedience. Composite scores based on this principal component analysis were calculated.

**Table 3:** **Factor. analysis (principal component analysis), varimax rotation on the items of the BFCRS^a^**.

	Negative factor	Stereotypy/mannerisms factor	Echo/perseveration factor	Excitement factor	Grimacing factor	Autonomic factor
Excitement	−0,320	0,509	0,070	**0,442**	0,070	−0,156
Immobility/stupor	**0,836**	−0,06	0,096	−0,135	−0,019	−0,143
Mutism	**0,837**	0,013	−0,068	−0,069	0,047	−0,177
Staring	**0,790**	0,140	0,086	−0,111	0,055	0,105
Posturing/catalepsy	**0,900**	0,007	−0,038	−0,037	0,006	−0,094
Grimacing	−0,065	0,142	−0,046	0,180	**0,637**	−0,065
Echopraxia/echolalia	−0,092	−0,209	**0,756**	−0,066	0,204	−0,247
Stereotypy	−0,074	**0,830**	0,096	0,008	−0,026	−0,005
Mannerisms	0,139	**0,608**	−0,071	0,215	0,053	0,138
Verbigeration	0,109	0,188	0,727	0,070	−0,149	0,270
Rigidity	**0,777**	0,069	0,146	0,096	−0,034	0,183
Negativism	**0,663**	0,115	0,033	0,137	0,490	0,257
Withdrawal	**0,665**	−0,129	−0,199	0,004	−0,219	−0,103
Impulsivity	0,112	0,492	0,134	**0,399**	0,192	0,030
Automatic obedience	−0,047	0,068	0,010	0,154	−0,068	**0,714**
Mitgehen	0,075	**0,688**	−0,018	−0,403	0,270	−0,127
Gegenhalten	**0,678**	−0,016	0,164	0,280	−0,041	0,053
Ambitendency	**0,650**	0,223	0,004	−0,069	0,545	0,068
Perseveration	0,243	0,348	**0,560**	−0,036	−0,403	0,09
Combativeness	0,006	0,068	−0,050	**0,728**	0,147	−0,018
Autonomic Abnormality	−0,033	−0,109	0,042	−0,285	0,053	**0,660**

*^a^Items waxy flexibility and grasp reflex were excluded from this analysis, because of the zero variance on these items*.

*Composite scores based on this principal component analysis (symptoms scores in bold) were calculated*.

### Clinical symptomatology

All patients completed the PANSS. Out of the total patient group, 88 (67.7%) had a PANSS-pos score higher than 14 reflecting a symptom state that was higher than dubious and 51 patients (39.2%) had at least mild psychotic symptomatology (i.e., a PANSS-pos score of 21 or higher). Similarly, all patients completed a YMRS: 29 patients (22.3%) had a score of 20 or higher, reflecting (hypo)manic symptomatology whereas only 34 patients (26.2%) had an absent of manic symptomatology (i.e., a maximum score of 6).

### To what extent is the catatonic symptomatology determined by the underlying diagnosis?

The total patient sample was divided in four groups: patients with a psychotic disorder (*n* = 67; 51.5%), patients with a mood disorder (*n* = 22; 16.9%; composed of 16 bipolar patients and 6 patients with a major depressive disorder), patients with a substance use disorder (SUD; *n* = 14; 10.8%), and patients with another diagnosis (patients-OD; *n* = 27; 20.8%).

**Figure 1:**
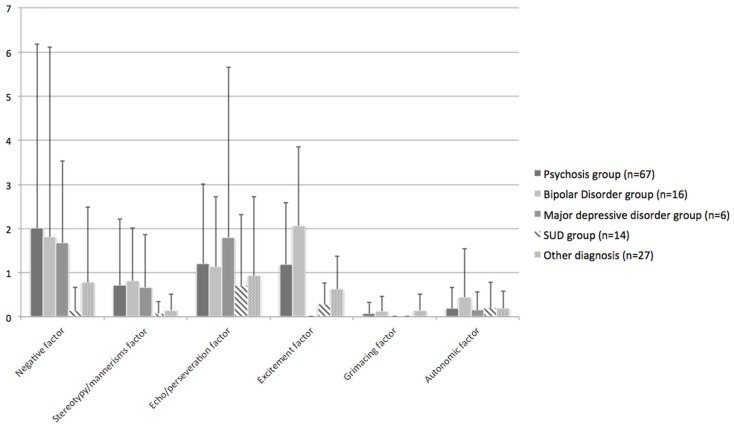
**Distribution of catatonic signs**.

Patients with a psychotic or mood disorder as a primary diagnosis had the most prominent catatonic symptom profiles (see Figure 1).

Compared to patients with a SUD or the patient-OD group psychotic patients tended to score higher on the stereotypy/mannerism symptom cluster (SUD: *p* = 0.044; patient-OD: *p* = 0.076), the negative symptom cluster (SUD: *p* = 0.069; patient-OD: *p* = 0.121), and on the excitement symptom cluster (SUD: *p* = 0.021; patient-OD: *p* = 0.063). No differences between the psychosis group and the combined mood disorder group could be seen. However, when only the bipolar patients entered analyses, these patients had significant more excitement symptoms (*p* = 0.015) than the patients with a psychotic illness, whereas, the latter group had significantly more excitement symptoms compared to the major depressive disorder group (*p* = 0.029). Very similar results were found after controlling for extrapyramidal symptomatology by use of the total score on the SAS. These results could mostly be explained by the fact that the SUD- and patient-OD groups hardly showed any catatonic symptomatology.

Psychotic symptomatology correlated strongly with excitement symptomatology (*r* = 0.528, *p* < 0.001) and to a lesser degree with the stereotypy/mannerisms symptom cluster (*r* = 0.289; *p* = 0.001) and the echo/perseveration symptom cluster (*r* = 0.185; *p* = 0.035). Similarly, manic symptomatology as assessed by the YMRS correlated strongly with the excitement symptom cluster (*r* = 0.596; *p* < 0.001) and to a lesser extent with the stereotypy/mannerisms symptom cluster (*r* = 0.277, *p* = 0.001). It should be noted that the PANSS-pos subscale and the YMRS strongly intercorrelated (*r* = 0.695; *p* < 0.011), which undoubtedly confounded these results.

A PANSS-dep was calculated including items depression, anxiety, and guilt feelings. Kontaxakis and colleagues found this subscale to intercorrelate with the Hamilton Depression subscale (23). PANSS-dep was inversely correlated with the grimacing factor (*r* = −0.288; *p* = 0.001) and tended toward an inversely correlation with the excitement factor (*r* = −0.170; *p* = 0.054), suggesting that depressive patients had these catatonic symptoms to a lesser degree than their non-depressed peers.

The total score on the SAS also correlated with the negative catatonia symptomatology (*r* = 0.350; *p* < 0.001) and with the echo/perseveration symptoms (*r* = 0.318; *p* < 0.001), which suggests that catatonic symptoms and extrapyramidal symptoms could not clearly be delineated from each other in our patient sample.

## Discussion

Out of the 130 patients that were admitted to an enclosed psychiatric ward, 101 patients (77.7%) had at least 1 symptom, whereas 66 patients (50.8%) had at least 2 symptoms when screened for catatonia symptoms, irrespective of the underlying diagnosis. In other words, catatonic symptomatology was highly prevalent in our patient population, although in most cases mildly. The most prevalent catatonic symptoms were excitement (*n* = 49; 37.7%), perseveration (*n* = 32; 24.6%), impulsivity (*n* = 31; 23.8%), and verbigeration (*n* = 31; 23.8%).

Our current findings demonstrate the presence of at least one symptom that is labeled as being catatonic by the BFCRS in most of the patients admitted to an enclosed psychiatric ward. In other studies, catatonia has been reported in 5–20% of acutely ill patients admitted to psychiatric units (9, 10, 18, 21, 24–26). In these studies, different criteria to diagnose catatonia were used, which renders a comparison between different studies on the prevalence of catatonia more difficult. For example, in the study of Lee, DSM-criteria were used to classify catatonia (24). When we used the latest DSM-criteria, only 16.9% of the patients (*n* = 22) could be considered as being catatonic. In the study of Ungvari, the diagnosis was made in the presence of four or more signs or symptoms with at least one having a score “2” or above on the BFCRS (26), which again, are more strict criteria than those used in our study. Fink and Taylor made their own diagnostic criteria with emphasis on the duration of the catatonic symptoms (22). Consequently, these divergent findings raise two interesting points. Depending on which criteria are being used, the more strict DSM-criteria versus the more liberal criteria suggested by Bush and colleagues (i.e., two items on the BFCRS), very different prevalence rates were found, which clearly emphasizes the shortcomings caused by a lack of clear-cut criteria (27). Of note, the DSM-5 criteria for catatonia appear to be even more strict than those of its predecessor, even if all 12 items, which were clustered in five categories in the DSM-IV can now be scored separately. This is mainly due to the fact that now three instead of two items have to be present. On the other hand, the high prevalence of symptoms using the BFCRS-criteria was mostly explained by the presence of mild symptomatology, whereas, more severe symptoms were present in a minority of our sample. Consequently, our results seem to point out that catatonic features, and more broadly psychomotor symptoms, may deserve a dimensional approach, much like cognitive symptoms associated with these psychiatric illnesses (27). It should also be noted that the most prevalent catatonic symptoms were not the strictly motor symptoms, which mostly seem associated with the traditional view on catatonia. Cognitive symptoms like perseveration and affective symptoms like excitement were the most prevalent and their validity and specificity as catatonic features should be questioned, especially in the more mild presentations. The unknown pathophysiology may contribute to the different views on catatonia. An unifying pathogenesis of catatonia that explains all motor, vegetative, and behavioral symptoms remains elusive. As a result, an unclear clinical concept of catatonia exists with the use of different diagnostical criteria and different rating scales to score catatonic symptomatology.

In our study, no significant differences in overall prevalence of catatonia between the psychosis group and the combined mood disorder group could be seen. Other studies also show that the syndrome is highly prevalent in both psychotic and mood disorders (17). Several studies found that the frequency of catatonia as part of schizophrenia varies with a range between 4 and 15% (5–8). Slightly higher prevalence rates have been shown in mood disorders with prevalence rates of 10–25% in bipolar disorder and up to 20% of patients with an unipolar depressive disorder (14–16, 22). However, again, different criteria for catatonia were used in these studies.

Different catatonia symptom profiles were found, depending on the underlying psychopathology. Psychotic patients tended to score higher on the stereotypy/mannerism symptom cluster, the negative symptom cluster, and the excitement symptom cluster compared to patients with a substance use disorder and patients with another diagnosis, but not compared to patients with mood disorders. In this line, psychotic symptomatology correlated strongly with excitement symptomatology and to a lesser degree with the stereotypy/mannerisms symptom cluster and the echo/perseveration symptom cluster. Similarly, manic symptomatology correlated strongly with the excitement symptom cluster and to a lesser extent with the stereotypy/mannerisms symptom cluster. Kraepelin already suggested that catatonia had a different symptomatology depending on the underlying pathology. Partly in line with our results, he described that negativism and mannerism were mainly associated to dementia praecox (4). Similarly, Schneider compared patients with catatonic (schizophrenic) and manic excitement, respectively and found that schizophrenic agitated patients displayed more blocking, waxy flexibility, stereotyped speech, mutism, and negativism (28). In a study of catatonic adolescents, automatic obedience and stereotypies were significantly more associated with schizophrenic than they were with non-schizophrenic catatonia (29). Finally, Krüger and colleagues found that catatonic chronic schizophrenia is mainly associated with catalepsy, waxy flexibility, and volitional disturbances such as automatic obedience and negativism, as well as mannerisms and abnormal involuntary movements such as grimacing, jerky movements, and stereotypies. In contrast, manic patients mainly displayed catatonic excitement, whereas, depressed patients were characterized by catatonic inhibition in terms of stupor, mutism, and rigidity (15). This was also in line with our findings, since symptoms of excitement and combativeness was significantly more present in the manic patients sample and significantly less in the depressed group, when compared to the psychotic patients sample.

Some limitations of our study should be pointed out. First, the impact of medication could be a confounding factor in our study. A vast number of patients were taking benzodiazepines at the time of testing, which could have masked more severe presentations of the catatonic syndrome. Another limitation of the study is the lack of a depression scale. To overcome this limitation, we used the PANSS-dep but a dedicated depression scale would have been more elegant. Moreover, the sample size was rather small, especially in some subgroups. Larger scale trials are needed to replicate our findings.

In conclusion, there was a high prevalence of catatonic symptomatology. Remarkably, there is an important difference in exact prevalence depending on the criteria being used, which makes it clear that we need clear-cut criteria. Another important finding is the fact that the catatonic presentation may vary depending on the underlying pathology, although an unambiguous delineation between these catatonic presentations cannot be made. Future research is needed to determine diagnostical criteria of catatonia, which are clinically relevant.

## Author Contribution

All authors met ICMJE criteria and all those who fulfilled those criteria were listed as authors. All authors had access to the study data and made the final decision about where to present these data.

## Conflict of Interest Statement

The authors declare that the research was conducted in the absence of any commercial or financial relationships that could be construed as a potential conflict of interest.
